# Graphene-Based Strain Sensing of Cementitious Composites with Natural and Recycled Sands

**DOI:** 10.3390/s23167175

**Published:** 2023-08-14

**Authors:** Uzma Bibi, Alireza Bahrami, Faisal Shabbir, Muhammad Imran, Muhammad Ali Nasir, Afaq Ahmad

**Affiliations:** 1 Civil Engineering Department, University of Engineering and Technology, Taxila 47050, Pakistan; 2 Department of Building Engineering, Energy Systems and Sustainability Science, Faculty of Engineering and Sustainable Development, University of Gävle, 801 76 Gävle, Sweden; 3 Mechanical Engineering Department, University of Engineering and Technology, Taxila 47050, Pakistan; 4 Department of Civil Engineering, The University of Memphis, Memphis, TN 38152, USA

**Keywords:** structural health monitoring, self-sensing, graphene nanoplatelets, recycled sand, cementitious composite, dispersion, ultrasonication, four-probe method

## Abstract

Structural health monitoring is crucial for ensuring the safety and reliability of civil infrastructures. Traditional monitoring methods involve installing sensors across large regions, which can be costly and ineffective due to the sensors damage and poor compliance with structural members. This study involves systematically varying the graphene nanoplatelets (GNPs) concentration and analyzing the strength performance and piezoresistive behavior of the resulting composites. Two different composites having natural and recycled sands with varying percentages of GNPs as 2%, 4%, 6%, and 8% were prepared. Dispersion of GNPs was performed in superplasticizer and then ultrasonication was employed by using an ultrasonicator. The four-probe method was utilized to establish the piezoresistive behavior. The results revealed that the compressive strength of mortar cubes with natural sand was increased up to a GNP content of 6%, beyond which it started to decline. In contrast, specimens with recycled sand showed a continuous decrease in the compressive strength. Furthermore, the electrical resistance stability was observed at 4% for both natural and recycled sands specimens, exhibiting linearity between the frictional change in the resistivity and compressive strain values. It can be concluded from this study that the use of self-sensing sustainable cementitious composites could pave their way in civil infrastructures.

## 1. Introduction

Cementitious composites are widely utilized in the construction of concrete structures due to their exceptional properties [1]. However, these materials possess certain drawbacks such as brittleness, susceptibility to cracking, and absence of electrical conductivity [2]. Concrete structures can experience degradation over time due to a variety of factors [3]. Rebar corrosion has been identified as a primary contributor to the deterioration of concrete structures [4,5]. Seismic events can cause extensive damage and failure of civil structures [6,7]. Moreover, with the increasing frequency of extreme weather conditions, such as hurricanes, tropical storms, and prolonged high temperatures, the health of concrete structures is expected to be significantly impacted [8]. Consequently, innovative approaches for detecting damage to civil infrastructures and mitigating potential degradation through timely maintenance interventions are imperative [9].

In recent decades, structural health monitoring (SHM) systems have been developed and used to continuously assess and monitor the condition of building systems [1,10,11]. These systems employ multiple sensors that detect parameters such as strain, displacement, and temperature to gather real-time data on the structural behavior [12]. Utilizing these data, owners and engineers can identify irregularities and perform necessary maintenance to enhance safety and durability [13]. However, traditional SHM solutions use a limited number of sensors spread across a large area of the structure, which can be costly, less durable, and less compatible with the host structure [11,14,15]. In this regard, cement-based self-sensing composites with inherent strain and damage-sensing properties offer a more practical and sustainable solution for monitoring the condition of civil infrastructures [16].

The strain-sensing ability of a material refers to its responsiveness to changes in volumetric electrical properties caused by variations in its strain condition [8,9]. When subjected to compression, the material experiences a decrease in electric resistance [13,17]. Conversely, when the material is under the tension, the resistance value increases owing to the dispersion of fillers [18]. As both effects are reversible within the material’s elastic range, the resistive property recovers to its original level upon unloading, as noted by [6]. However, when subjected to plastic deformations, the electrical resistance undergoes irreversible changes [19]. While the former property can be leveraged for strain-sensing applications, the latter can be employed for damage-sensing purposes [20].

Self-sensing materials offer numerous advantages over conventional strain sensors, including superior reliability, compatibility with the cement matrix, and spatially explicit measuring capacity [21,22]. Research into the behavior of self-sensing cementitious composites with various fillers has been ongoing since the early 1990s [23,24], with a focus on enhancing the versatility of the base material through the use of carbon fiber, carbon nanofiber, and carbon nanotubes [5,25,26]. Graphene, which was discovered in 2004 as a 2D nanoscale material for composite applications, has gained significant attention thanks to its ultra-high specific surface area and resulting increased contact area with the host material [27,28]. While the high cost and dispersion challenges associated with graphene have been noted as drawbacks [29], graphene nanoplatelets (GNPs) have emerged as a promising alternative due to their exceptional material qualities, low cost, and ease of production [30]. GNPs possess the necessary mechanical and physical properties for a fraction of the cost of carbon nanotubes (CNTs), making them an attractive option for the industry [31,32]. Graphene has emerged as a viable material for sensing applications owing to its extraordinary characteristics [33,34,35].

Recycled materials have gained importance in recent times as the world is shifting toward sustainable construction [15]. Recycled materials remarkably reduce the demand for natural resources [36]. Demolition waste is leading the world toward serious environmental changes [37]. There is a current immediate need to conserve natural resources. The use of recycled materials not only minimizes waste pollution, but it is also cost-effective [37,38]. Sand is continuously being depleted worldwide due to its use in concrete construction [39]. Too much extraction of sand from rivers and other sources creates an imbalance in the environment [22]. Recycled sand is preferred over natural sand because it provides a sustainable approach to lowering natural sand demand, minimizing environmental impacts, conserving resources, and promoting circular economy standards in the construction industry.

For this reason, the understanding of the behavior of cementitious composites containing different sustainable materials is still under investigation. Additionally, despite the noticeable efforts made to advance intrinsically self-sensing cementitious materials incorporating various nanofillers, limited efforts have been conducted on the properties of cementitious composites integrating GNPs in conjunction with sustainable resources. As a result, there is a need for further research on assessing the performance of cementitious composites with virgin and recycled aggregates for the goal of monitoring structural health.

In the sections that follow, Section 2 focuses on the materials and methods, Section 3 describes the results and provides the discussion, and Section 4 presents the conclusions obtained from the experimental study reported in the prior sections.

## 2. Materials and Methods

### 2.1. Preparation of Specimens 

**Figure 1:**
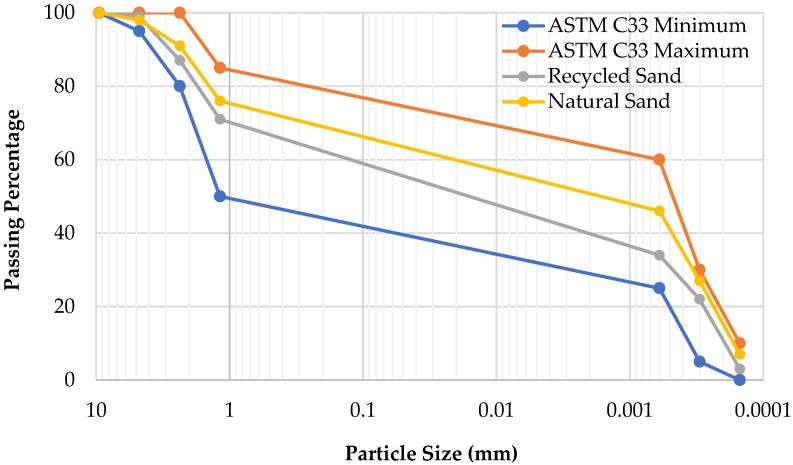
Gradation curves for natural and recycled sands.

Natural sand is a standardized reference material that allows for reliable comparisons and analysis in a variety of investigations. In this research, natural sand was obtained from a riverbed. In addition, recycled sand obtained from concrete laboratory waste provided a sustainable option that is in line with ecologically reliable practices. Portland cement was utilized as a binder along with sand. Different sieves were used, i.e., #4, #8, #16, #30, #50, and #100, along with the pan. The sand that passed through a #16 sieve and retained on a #100 sieve was utilized in a ratio of 1:3. The results of the sieve analysis of natural and recycled sands are shown in Figure 1. GNPs purchased from ACS Material (USA) were incorporated into the mixture. These GNPs consist of stacks of graphene with thicknesses ranging from 2 to 10 nanometers and widths ranging from 2 to 7 μm [40]. Table 1 provides information about the characteristics of GNPs.

#### 2.1.1. Dispersion of GNPs

An ultra-superplasticizer (SP) 675 [41] was employed to enhance the workability and dispersion of GNPs in mortar. The SP/GNPs ratio was established at 1:8, based on the previous research [42]. However, it was observed that the quantity of SP used in the experimental batches of mortars with 8% GNPs was inadequate to achieve the desired mixed workability. The increased surface area of GNPs relative to Portland cement requires a larger amount of SP for the dispersion. Consequently, the SP dosage for 8% GNPs was modified, resulting in an increased dose of SP in mortar with an 8% GNP batch.

#### 2.1.2. Ultrasonication Process

**Figure 2:**
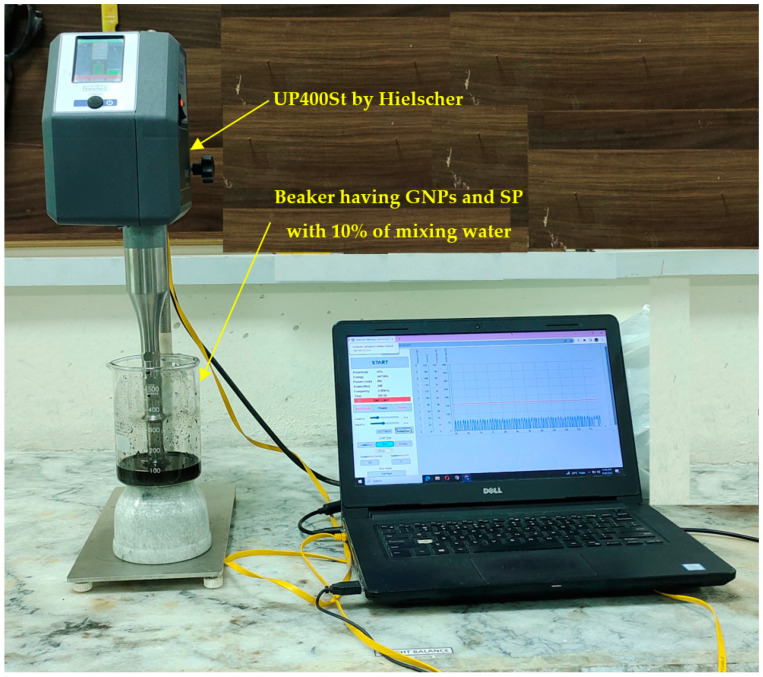
Application of UP400St ultrasonicator, Hielscher, Germany for ultrasonication.

The ultrasonication process employed to disperse GNPs utilized a UP400St model ultrasonicator manufactured by Hielscher, Germany. The duration of ultrasonic treatment was approximately 30 min. The ultrasonic energy was delivered to the suspensions by means of a probe, operating at 60% of its maximum power, with an amplitude of 40% and a pulse of 50%. The frequency and amplitude of the ultrasonic waves were recorded, and the setup is illustrated in Figure 2.

### 2.2. Methodology 

**Figure 3:**
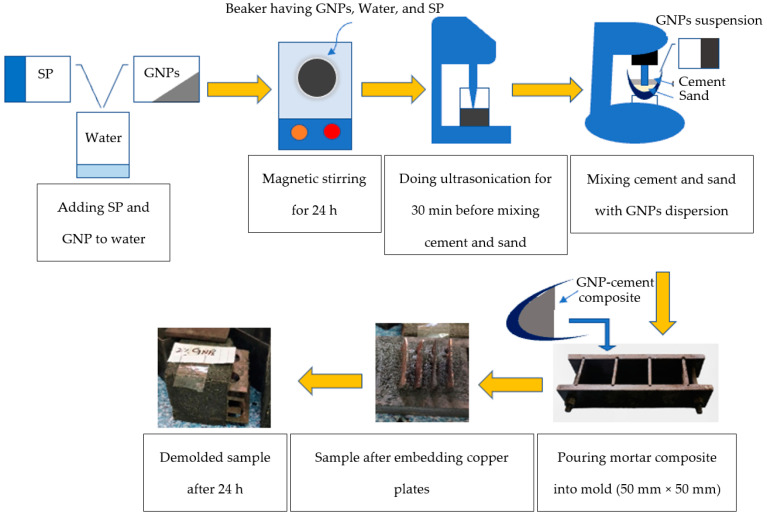
Schematic diagram showing fabrication process for mortar composites.

The methodology adopted for the fabrication process of the mortar composite is displayed in Figure 3. The process involved weighing and mixing GNPs with 10% of mixing water along with SP. The solution was subsequently stirred using a magnetic stirrer for 24 h. Ultrasonication was done for approximately 30 min. Following ultrasonication, cement and sand were mixed for 10 min at 110 rpm in a mortar mixer. Next, the dispersed GNP solution was added to the dry ingredients for 10 s, and the mixer speed was increased to 300 rpm for an additional 10 min blending. The resulting mixture was then poured into molds for the subsequent analysis.

A series of ten cement mortar batches were produced, each containing varying quantities of GNPs. Out of ten, five batches were made using natural sand, while the other five were made with recycled sand. Each batch was prepared in accordance with ASTM C109, and the resulting specimens had dimensions of 50 mm × 50 mm × 50 mm with a water-to-cement ratio of 0.48. GNPs were added to the conventional mortar specimens in five different proportions (0%, 2%, 4%, 6%, and 8% by weight of cement). The specimens were then cured at the room temperature and wrapped in plastic sheets.

**Figure 4:**
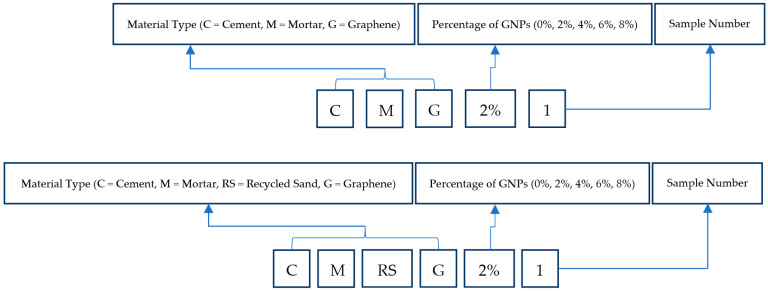
System used to assign labels to different specimens.

The labeling system assigned to the different specimens when subjected to the different tests under different loadings is demonstrated in Figure 4. Cement mortar with natural sand having 0%, 2%, 4%, 6%, and 8% graphene are represented as CMG0%, CMG2%, CMG6%, and CMG8%, respectively. Moreover, cement mortar with recycled sand having 0%, 2%, 4%, 6% and 8% graphene are represented as CMRSG0%, CMRSG2%, CMRSG6%, and CMRSG8%, respectively.

A comprehensive record of the tests carried out to establish the material and piezoresistive properties of mortar is represented in Table 2. Various percentages of GNPs, ranging from 0% to 8%, were employed to determine the piezoresistive behavior of mortar. This entailed conducting compression and piezoresistive tests on mortar cubes fabricated with both natural and recycled sands.

**Figure 5:**
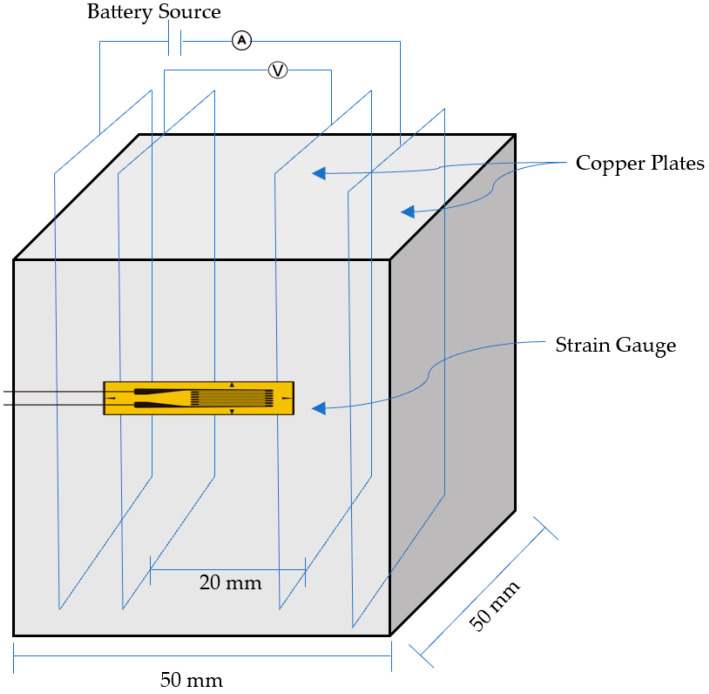
Resistivity measurement scheme with cyclic loading.

To facilitate the measurement of piezoresistivity, each specimen was implanted with four copper plates acting as electrodes. Copper plates with 0.4 mm thick were embedded as electrodes with the probes spaced 20 mm apart [43]. Figure 5 depicts the specimen together with the strain gauge and electrode arrangement. A strain gauge was utilized to measure strain, and electrodes were implanted to measure the electrical resistance. The current was supplied to the outside electrodes, and the voltage was measured at the inner electrodes. After smoothing the surface of the specimens with sandpaper, the gauge was connected with the glue provided with the strain gauges. The gauge was mounted in the center of the face, parallel to the load axis, between the inner electrodes.

#### 2.2.1. Compressive Strength Testing

In order to investigate the impact of GNPs on the compressive strength, a series of compressive strength tests were done on cube specimens. The experimental design included a control group of plain mortar specimens. The testing procedure was performed using an MCC8 machine, CONTROLS, Milano, Italy, following the standard procedure outlined in ASTM C109 with a loading rate of 1000 N/s.

#### 2.2.2. Piezoresistive Testing

The specimens were subjected to a curing time of 28 days, after which they were subjected to oven-drying at 105 °C for 24 h to reduce the moisture content and minimize its effect on subsequent measurements. To further minimize the effect of the contact resistance, the piezoresistive measurements were carried out using a 4-probe approach. In this approach, two multimeters were used to evaluate the resistance value. A direct current (DC) of 12 V was supplied via a power source, with one meter connected to the external probe and power source to measure the current, and the second multimeter connected to the interior probes to detect voltage. Cyclic load testing was conducted with measurements of the stress, strain, current, and voltage taken simultaneously. Resistance over time was calculated by using the Ohm’s law, and resistivity was calculated on the basis of resistance by employing the following formula:

(1) $$\rho =\frac{RA}{L}$$

where

ρ—resistivity;
R—resistance value;
A—area (25 mm × 50 mm);
*L*—length (25 mm).

The relationship between the frictional change in resistivity (FCR) and strain was measured. To quantify FCR, temporal variation in resistivity was divided by the initial resistivity value, as expressed by the following equation:

(2) $$FCR=\frac{∆\rho }{\rho_{0}}$$

where

FCR—change in resistivity;
∆ρ— change in resistivity over time;
$\rho_{0}$—initial resistivity value.

**Figure 6:**
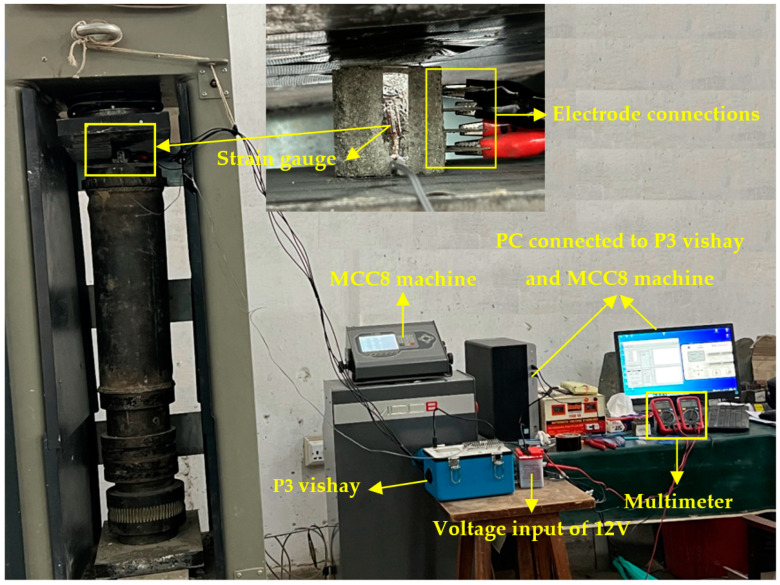
Test setup for piezoresistive testing of specimens.

Overall, these measures were taken to ensure accurate and reliable measurements of resistance, with steps taken to minimize the impact of the moisture and contact resistance on the results. The compression testing equipment utilized in this study was operated by an MCC8 controller. To minimize the effects of strain and resistance, a 5 kN preload was applied to the specimens and held for 1 min duration. To conduct piezoresistive measurements, the specimens were subjected to a cyclic load at a rate of 200 N/s. The experimental program used for doing the piezoresistive test is indicated in Figure 6.

**Figure 7:**
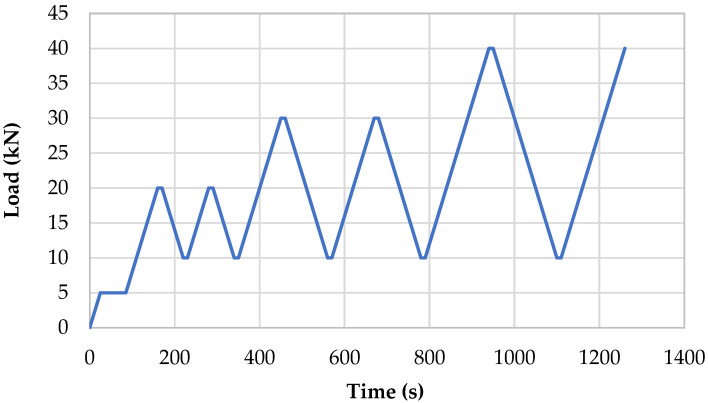
Load cycles for piezoresistivity testing.

In Figure 7, the load cycles applied to the specimens are shown. Each load cycle was repeated followed by a subsequent load increment [43]. The cyclic sequence demonstrates that the specimens were initially subjected to a 5 kN preload, followed by loading up to 20 kN and holding for 10 s. The load was subsequently reduced to 10 kN and held for 10 s. The second cycle was identical to the first. On the third and fourth cycles, the load was increased to 30 kN, and on the fifth and sixth cycles, it was increased to 40 kN. The experiment was performed at the room temperature.

## 3. Results and Discussion

### 3.1. Compressive Strength of Specimens Having Natural Sand

This section provides a comprehensive summary of the compressive strength analysis of cement mortar using natural sand (CMG series) with the graphene percentages of 0%, 2%, 4%, 6%, and 8%.

After a period of 28 days, the compressive strength of the cubes was evaluated utilizing a compression testing machine. The piezoresistive tests were done concurrently with the strength performance examination at the age of 28 days. The strength performance was determined using CMG0% and CMRSG0% as references, since they were built of traditional mortar. An outcome exceeding 100% signifies better performance than that for the reference specimens. The strength performance was estimated employing the following equation:

(3) $$Strength\;performance\;\left(\%\right)=\frac{Strength\;of\;mix}{Strength\;of\;control\;mix}\times 100$$

**Figure 8:**
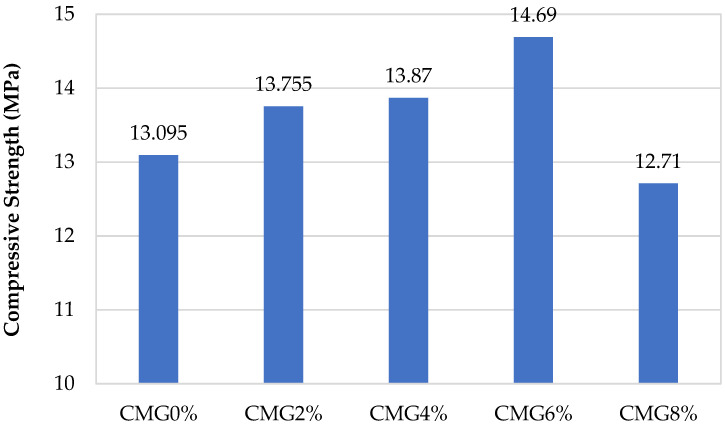
Compressive strength of specimens having natural sand with different percentages of GNPs.

Figure 8 illustrates the compressive strength analysis, and it reveals a significant correlation between the GNP percentages and the compressive strength of the specimens. The evaluation indicated that the specimens labeled as CMG2%, CMG4%, and CMG6% had superior performance compared to the control mix. Notably, the specimens containing CMG2%, CMG4%, and CMG6% exhibited considerable increases in the compressive strength with the percentage increments of 5.04%, 5.92%, and 12.18%, respectively. Conversely, CMG8% gave a decline of 3.09% in the compressive strength when compared to the control specimen (CMG0%).

The compressive strength was improved when the GNP content was increased to a particular level of 6% before it started to decline. Filling of the cement mortar’s micropores might be the cause for the increased strength. GNPs acted as a filler, filling the gap between cement and sand and increasing the strength. This pattern persisted up to a particular percentage of GNPs, known as the threshold value. If the value were higher than the threshold, the fillers would become saturated, and appropriate textural binding of composites could not be performed, which lowered the strength. When the GNP concentration exceeded 6% replacement by weight of cement, more segregation and lower strength were observed.

### 3.2. Piezoresistive Behavior of Specimens Having Natural Sand

An investigation was carried out on the piezoresistive behavior of graphene at various percentages when subjected to cyclic loading. The piezoresistive behavior varied from mix to mix and depended on the quantity of GNPs employed under cyclic compressive stress. The GNP particles at a certain percentage come into close contact with each other, thus forming conductive paths in the entire body of the composites. It is evident from the behavior of all mix compositions that FCR was reduced while loading and enhanced upon unloading. This behavior may be explained by the fact that loading brings the particles closer together, creating more paths for current to flow through.

**Figure 9:**
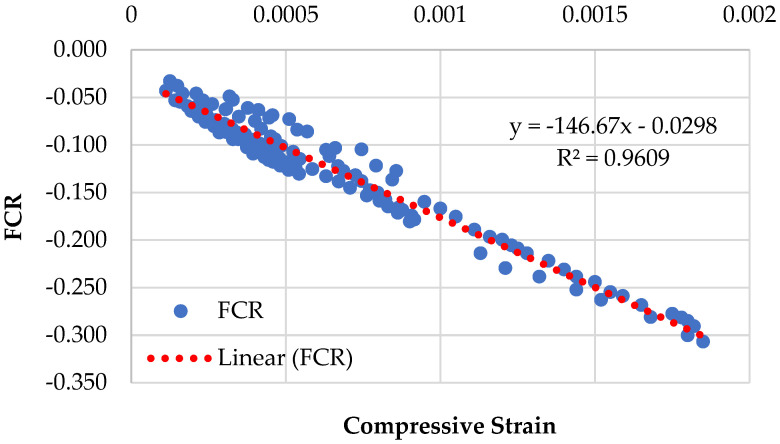
Piezoresistive behavior of mix (CMG series) containing 2% GNPs.

**Figure 10:**
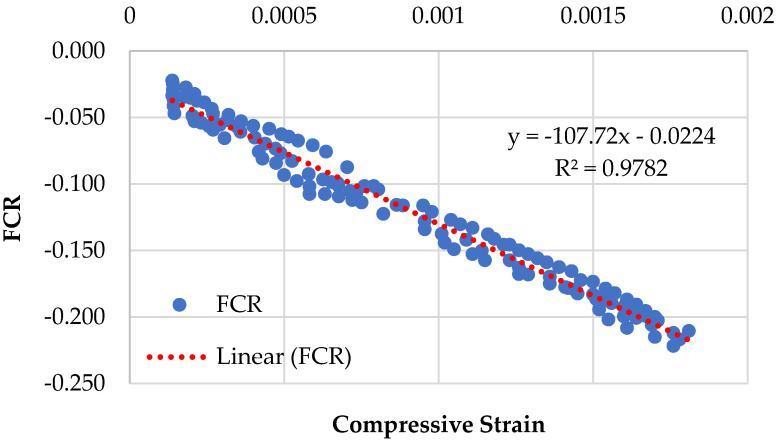
Piezoresistive behavior of mix (CMG series) containing 4% GNPs.

**Figure 11:**
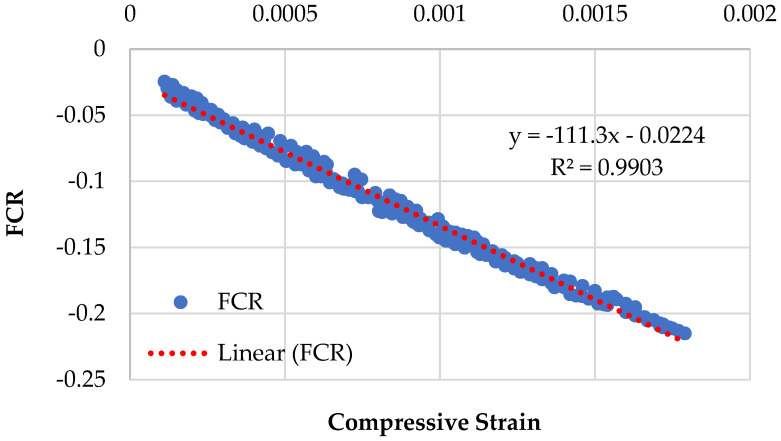
Piezoresistive behavior of mix (CMG series) containing 6% GNPs.

**Figure 12:**
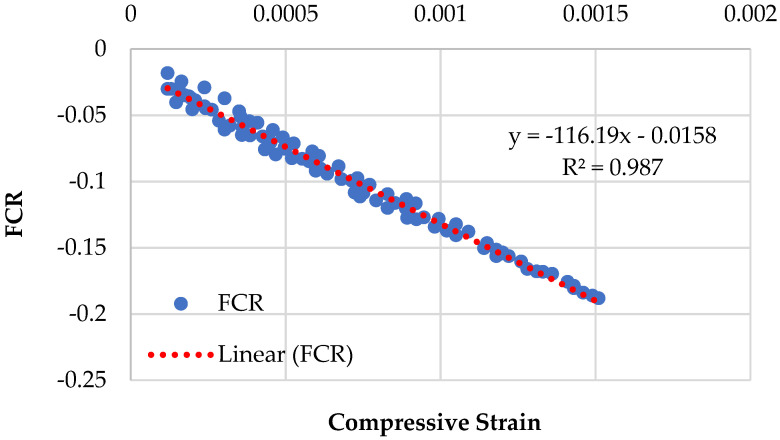
Piezoresistive behavior of mix (CMG series) containing 8% GNPs.

Graphical representations of the piezoresistive behavior of the different compositions, namely CMG2%, CMG4%, CMG6%, and CMG8%, are displayed in Figure 9, Figure 10, Figure 11 and Figure 12. The standard deviations of CMG2%, CMG4%, CMG6%, and CMG8% were calculated as 0.069, 0.058, 0.056, and 0.060, respectively. The low standard deviations in CMG4% and CMG6% series advocate less noise in the readings, showcasing its potential for applications requiring precise sensing and measurement. In contrast, the piezoresistive behavior of the CMG8% specimen indicated increased noise in the results. The CMG2% specimen, which contained a suboptimal amount of GNP, generated more noise, affecting the accuracy of measurements. Similarly, CMG8% exhibited the piezoresistive behavior, but there was noise in the data and the strength was considerably reduced in those specimens. Because the GNP concentration in CMG8% was higher than the optimal amount, the binding between the particles weakened, resulting in fractures and noise in the piezoresistive results.

The purpose of the linearity tests is to assess whether the relationships between the dependent and independent variables are linear. A linear connection between the dependent and independent variables is required for effective research. *R*^2^ values represent the linearity in the data when a straight line is fitted to it. To evaluate the linearity of the data, the *R*^2^ values were calculated by fitting a perfect line. The *R*^2^ value for the CMG6% specimen was seen to be closer to 1, implying minimal noise in the results for CMG6%.

The results of the linearity tests between FCR and the compressive strains for the piezoresistive behavior of the mixes composition are depicted in Figure 9, Figure 10, Figure 11 and Figure 12. For example, for the mix CMG2% (Figure 9), FCR is given by:

(4) $$FCR=-146.67{ɛ}_{c}-0.0298\;\;with\;R^{2}=0.9609$$

where ε*_c_* denotes the concrete compressive strain.

It was witnessed that the relationship between FCR and the compressive strains of concrete is linear. The mix CMG6% with *R*^2^ = 0.9903 had the highest prediction accuracy, indicating that it had the best linear connection between FCR and the compressive strains with the lowest standard deviation. Further analysis is presented in Section 3.5.1 on the covariance of the regression equations pertaining to different CMG percentages.

### 3.3. Compressive Strength of Specimens Having Recycled Sand

This section provides a comprehensive summary of the compressive strength of cement mortar utilizing recycled sand with graphene percentages of 0%, 2%, 4%, 6%, and 8%. Here, the compressive strength of the mortar specimens containing recycled sand was examined.

The compressive strength results showed a decrease in the strength for CMRSGs. This may be attributed to the rough particle size distribution of recycled sand that affected the packing density, resulting in voids. Furthermore, the presence of already hydrated cement residues on the surface of recycled sand led to excess water demand, which weakened the mortar specimens. Therefore, due to the presence of large voids in the cementitious composite in recycled sand and increased water demand, a decrease in the compressive strength was observed in the CMRSG specimens.

**Figure 13:**
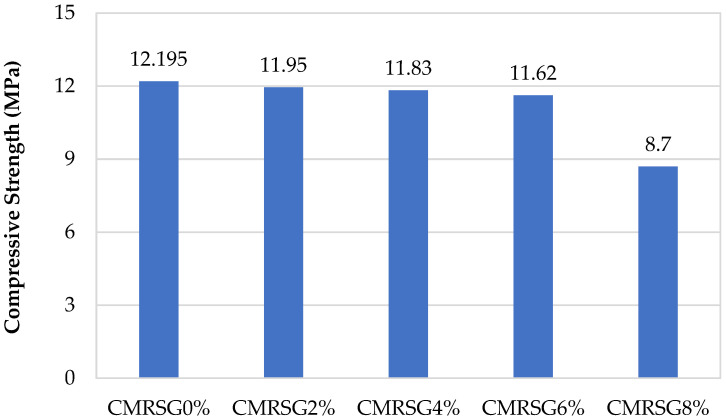
Compressive strength of specimens having recycled sand with different percentages of GNPs.

CMRSG2%, CMRSG4%, CMRSG6%, and CMRSG8% exhibited the compressive strength reductions of 2.01%, 2.99%, 4.72%, and 28.66%, respectively, compared to CMRSG0%, as illustrated in Figure 13.

### 3.4. Piezoresistive Behavior of Specimens Having Recycled Sand

**Figure 14:**
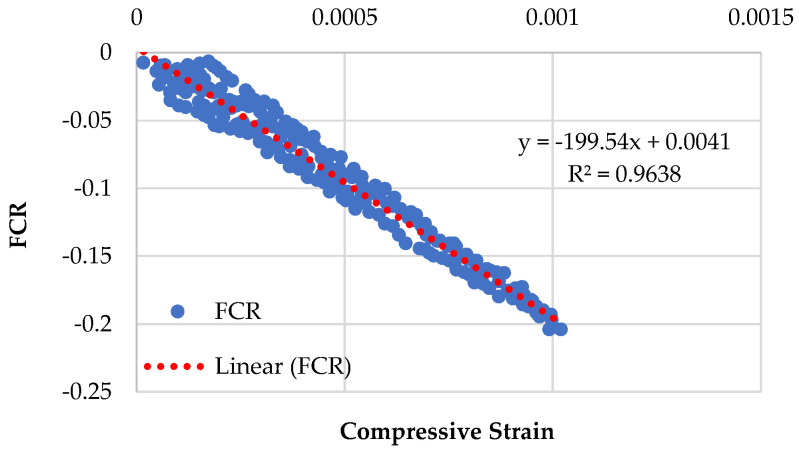
Piezoresistive behavior of mix (CMRSG series) containing 2% GNPs.

**Figure 15:**
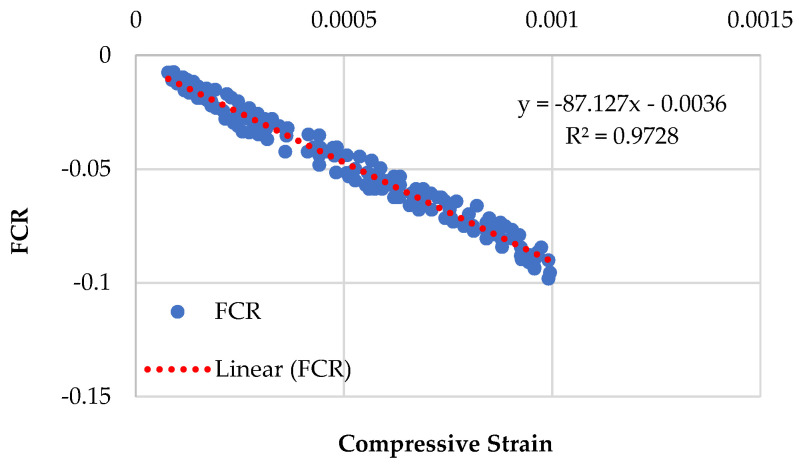
Piezoresistive behavior of mix (CMRSG series) containing 4% GNPs.

**Figure 16:**
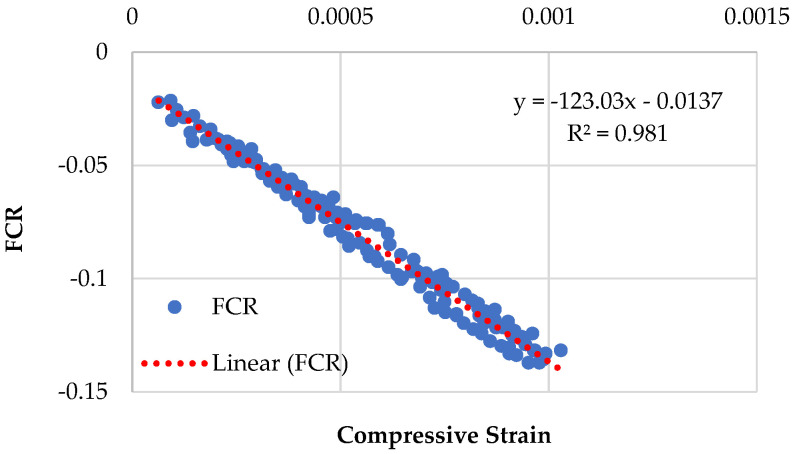
Piezoresistive behavior of mix (CMRSG series) containing 6% GNPs.

**Figure 17:**
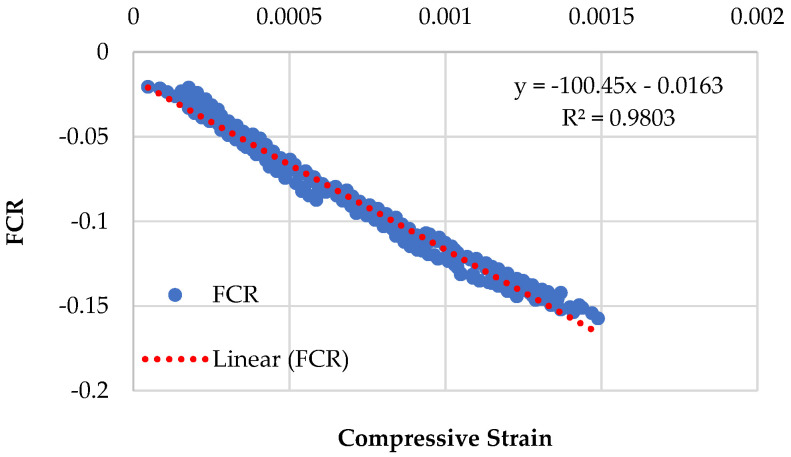
Piezoresistive behavior of mix (CMRSG series) containing 8% GNPs.

Figure 14, Figure 15, Figure 16 and Figure 17 demonstrate the piezoresistive behavior of the specimens containing recycled sand, as CMRSG2%, CMRSG4%, CMRSG6%, and CMRSG8%, respectively. The piezoresistive behavior of the specimens was evaluated. The standard deviations for the CMRSG2%, CMRSG4%, CMRSG6%, and CMRSG8% specimens were found to be 0.055, 0.024, 0.033, and 0.039, respectively. It was noted that the standard deviation values for the CMRSG specimens were comparatively lower than their corresponding values for the CMG specimens. Also, the compressive strength values for the CMRSG series were lower than their corresponding values for the CMG series. Therefore, fewer points were obtained during the experiments with the CMRSG specimens, which might be the reason for the lower standard deviation values. The results indicated that CMRSG4% exhibited reduced noise compared to the specimens with the other percentages. In contrast, CMRSG2%, CMRSG6%, and CMRSG8% exhibited higher noise levels, because of the creation of gaps and voids. Further analysis is given in Section 3.5.1 on the covariance of the regression equations pertaining to different CMRSG percentages.

### 3.5. Comparative Analysis

**Figure 18:**
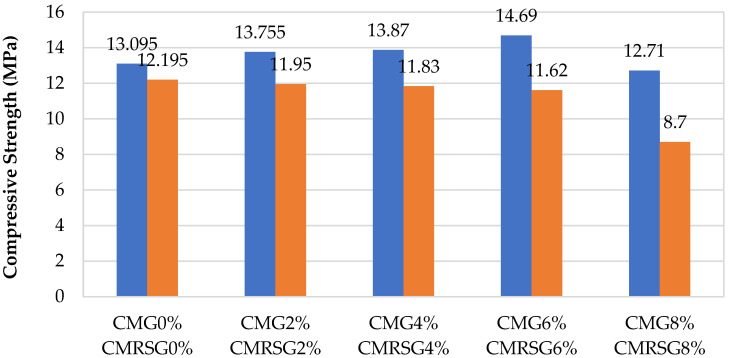
Comparison of compressive strengths of CMG and CMRSG series having different percentages of GNPs.

The compressive strengths were compared for both natural and recycled sands specimens, as displayed in Figure 18. It can be mentioned that recycled sand gave lower strengths compared to natural sand. The maximum compressive strength of the natural sand specimens was found to be for CMG6% as 14.69 MPa, whereas the maximum compressive strength of the recycled sand specimens was achieved for CMRSG2% as 11.95 MPa. However, the recycled sand specimens’ maximum compressive strength was around 18.65% lower than that of the natural sand specimens.

The comparison of the piezoresistive behavior of the specimens with natural and recycled sands showed a similar trend as seen in the case of their compressive strengths. The *R*^2^ values were obtained for the relationship between the compressive strains and FCR. The *R*^2^ value of 0.9903 was obtained for CMG6% as compared to the *R*^2^ value of 0.981 for CMRSG6%. Closer value to 1 achieved for the specimen with natural sand (CMG6%) implies better piezoresistive behavior of natural sand over recycled sand.

#### 3.5.1. Analysis of Covariance of CMG and CMRSG Series

**Figure 19:**
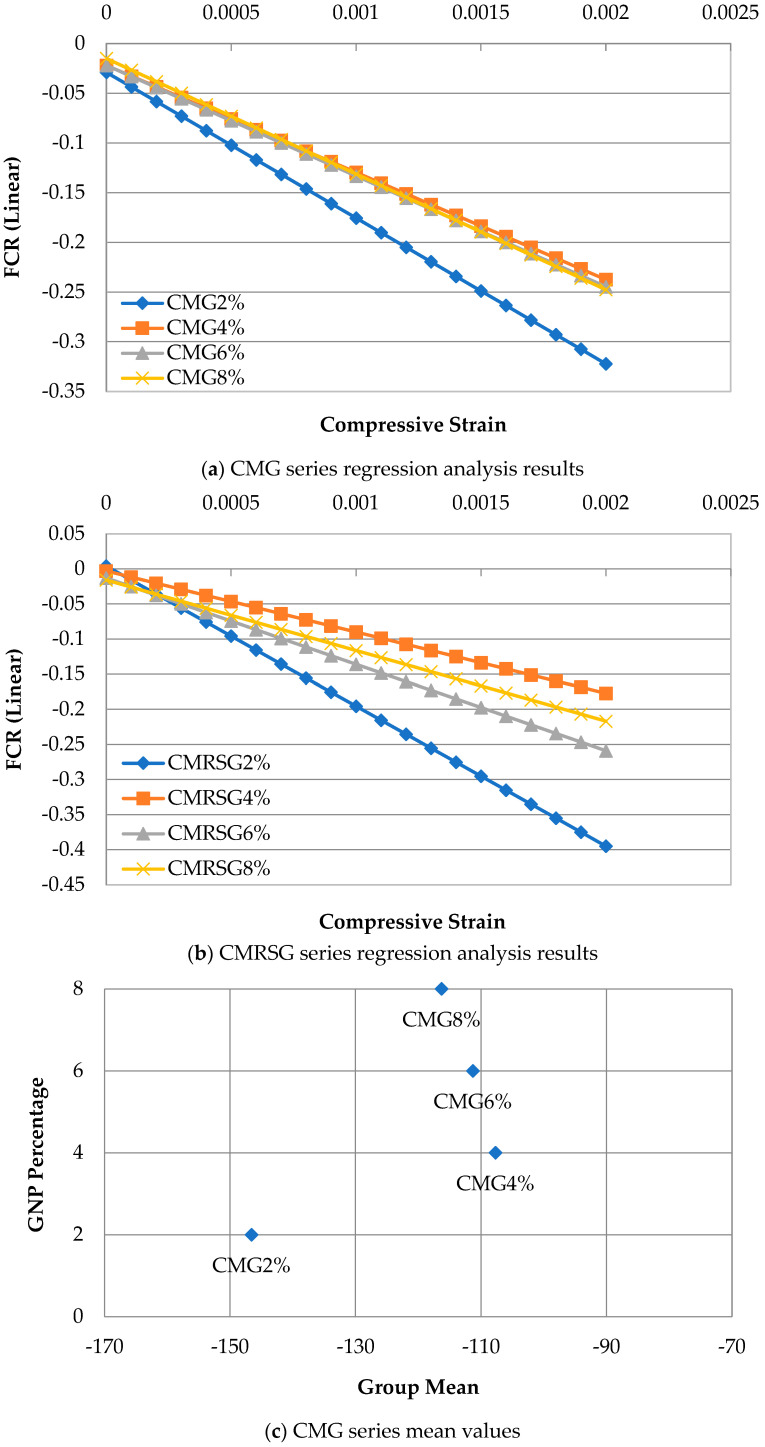

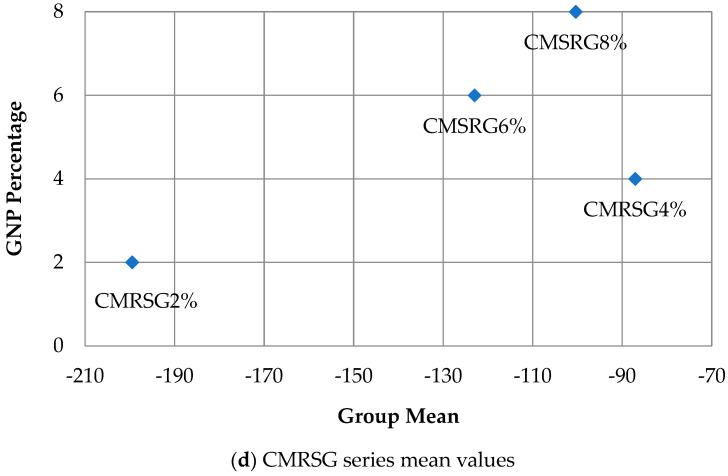
Covariance analysis for CMG and CMRSG series.

This section presents the covariance analysis of the regression equations. The covariance analysis of the regression equations of the CMG and CMRSG series are illustrated in Figure 19a,b, whereas their mean values are presented in Figure 19c,d. The findings revealed that the piezoresistivity of the composite mortar underwent significant changes only up to a 4% addition of GNP. Beyond this threshold, the regression analysis results for both CMGs and CMRSGs were observed to be very similar, as depicted in Figure 19a,b. Additionally, the mean values for the 4%, 6%, and 8% GNP concentrations were also found to be closely clustered together, in contrast to the 2% GNP concentration, as displayed Figure 19c,d. This finding signifies that beyond 4% GNPs, there was minimal impact on the conductivity. These results suggest that the conductivity paths in the composite mortar were reasonably established in the presence of 4% GNPs, and further additions of GNPs did not considerably affect the results.

In summary, the analysis of covariance revealed that the conductivity of the composite mortar was improved from 2% addition of GNPs to 4% addition of GNPs. The subsequent increases in the GNP concentrations did not noticeably alter the conductivity. Therefore, it is recommended that 4% GNPs can be used for cost saving instead of higher percentages of GNPs.

Research has been conducted in the past few decades on electrically conductive cement composites incorporating different percentages of GNPs. For comparison purposes, Table 3 summarizes the studies investigating the effects of varying GNP concentrations on the properties of the composites [44,45,46,47,48]. It can be noted that the previous studies also recommended the GNP percentages from 2.5% to 6%, close to those witnessed in this study.

**Figure 20:**
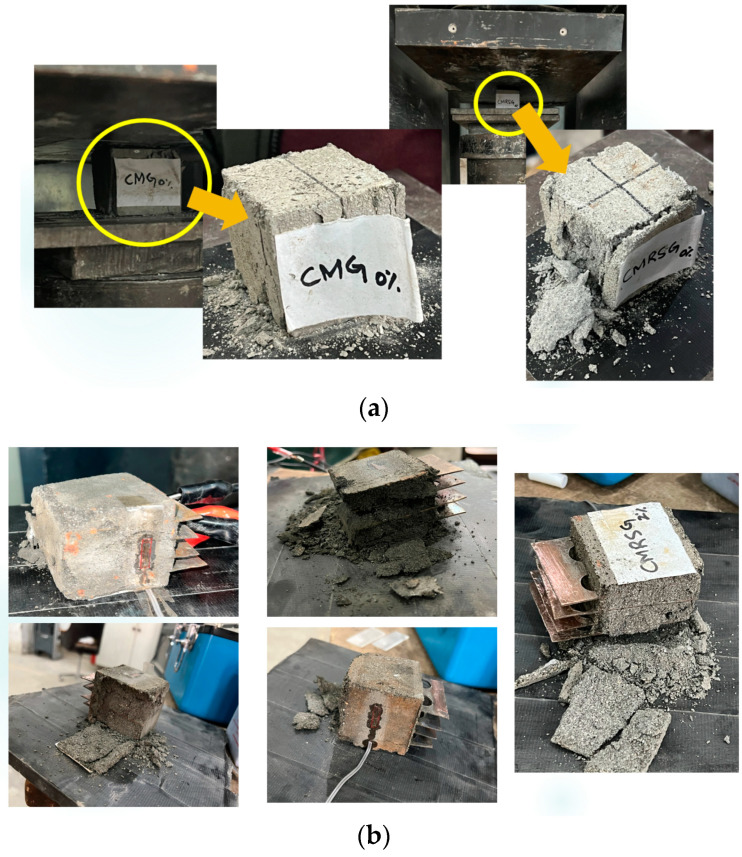
Specimens after failure that were subjected to piezoresistive testing: (**a**) CMG series; (**b**) CMRSG series.

Moreover, the crack pattern analysis on the specimens with recycled and natural sands was done, advancing understanding their behavior and characteristics. The crack pattern investigations involved subjecting both types of the specimens to compressive loading conditions and examining the resulting crack formations, as shown in Figure 20. The study uncovered distinct differences in the crack patterns between the specimens with recycled and natural sands. The specimens with natural sand, which served as the reference material, demonstrated a more uniform crack pattern. However, the specimens with recycled sand, sourced from concrete waste, exhibited crack patterns that were influenced by the presence of impurities and irregularities within the specimens. These impurities and irregularities acted as stress concentration points, leading to the formation of localized cracks in the specimens. It can be stated that the absence of impurities and irregularities in the specimens with natural sand contributed to a more consistent distribution of cracks throughout the specimens.

Contrasting the crack patterns have implications for the mechanical properties of the specimens containing recycled and natural sands. The presence of localized cracks in the specimens with recycled sand affected the overall compressive strength, as it could compromise the integrity of the material. In contrast, the more uniform crack distribution in the specimens with natural sand suggested better load-bearing capabilities and potentially improved long-term performance.

## 4. Conclusions

The primary goal of this research was to evaluate the compressive strength and self-sensing ability of cement composites with natural and recycled sands in pursuit to use sustainable materials in construction. Various percentages of GNPs were employed from 0% to 8%. Based on the obtained results, the following conclusions can be drawn:

- GNP as a partial replacement for cement enhanced the compressive strength and self-sensing capabilities when compared to the control specimens.
- The compressive strength of mortar cubes with natural sand was increased with increasing the content of GNPs. However, after a certain ratio, the strength began to decline. The strength performance was examined based on the control specimens, and the results for the CMG series with 2%, 4%, 6%, and 8% GNPs showed increases of 5.04%, 5.92%, and 12.18%, and a decrease of 3.09% in the strength, respectively.
- On the other hand, the specimens with recycled sand demonstrated a continuous decrease in the compressive strength. The strength performance for the CMRSG series with 2%, 4%, 6%, and 8% GNPs indicated decreases of 2.01%, 2.99%, 4.72%, and 28.66% in the strength, respectively. The decrease in the strength could be attributed to the creation of gaps or voids.
- The piezoresistivity of the composite mortar experienced noticeable changes only up to a 4% addition of GNPs. Beyond this percentage, the regression analysis results for both CMGs and CMRSGs were seen to be very similar, which was supported by the findings of the covariance analysis of the regression equations. The addition of GNPs enhanced the ability to monitor structural health.
- The crack patterns displayed that more uniform cracks were obtained for the CMG series compared to the CMRSG series.

These findings emphasize the importance of considering the quality and characteristics of sand sources. Understanding the crack patterns and their underlying causes can guide in ensuring the use of suitable sand sources and developing sustainable approaches to waste management in the construction industry.

## Figures and Tables

**Table 1:** GNPs characterization [40].

Appearance	Black/Gray Powder
Diameter	2–7 μm
Thickness	2–10 nm
Specific surface area	20–40 m^2^/g
Electrical conductivity	800–1100 S/cm
Carbon content	>99%
Apparent density	0.06–0.09 g/mL
Water content	<2 wt.%
Residual impurities	<1 wt.%
Particle size distribution	D10 = 13.56 μm
	D50 = 48.93 μm
	D90 = 122.2 μm

**Table 2:** Performed tests to establish compressive strength and piezoresistive behavior.

No.	Specimen ID	Performed Test	Percentage of GNP	Filler Material
1	CMG0%	Compression	0%	Natural Sand
2	CMG2%	Compression and Piezoresistive	2%	Natural Sand
3	CMG4%	Compression and Piezoresistive	4%	Natural Sand
4	CMG6%	Compression and Piezoresistive	6%	Natural Sand
5	CMG8%	Compression and Piezoresistive	8%	Natural Sand
6	CMRSG0%	Compression	0%	Recycled Sand
7	CMRSG2%	Compression and Piezoresistive	2%	Recycled Sand
8	CMRSG4%	Compression and Piezoresistive	4%	Recycled Sand
9	CMRSG6%	Compression and Piezoresistive	6%	Recycled Sand
10	CMRSG8%	Compression and Piezoresistive	8%	Recycled Sand

**Table 3:** GNP percentages recommended for SHM.

Reference	GNP Percentage Recommended for SHM
Du and Dai Pang [44]	6.4% (by weight)
Du et al. [45]	2.5% (by weight)
Jiang et al. [46]	2% (by volume)
Liu et al. [47]	2.4% (by weight)
Sun et al. [48]	5% (by weight)
Present study	4% (by weight)

## Data Availability

Not applicable.
